# A Dictionary of Practical Surgery, &c.

**Published:** 1839-04

**Authors:** 


Art. III.—1. *A Dictionary of Practical Surgery: comprehending all the most interesting Improvements from the earliest times down to the present period, &c. &c. The Seventh Edition, revised, corrected, and enlarged.* By Samuel Cooper, Professor of Surgery in University College, London, &c. &c.—*London,* 1839. 8vo. pp. 1518.

2. *Lexicon Medicum, or Medical Dictionary: containing an Explanation of the Terms in Anatomy, Botany, Chemistry, Materia Medica, Pharmacy, Physiology, Practice of Physic, Surgery, &c. &c.* By the late Robert Hooper, m.d. f.l.s. *The Seventh Edition, revised, corrected, and enlarged.* By Klein Grant, m.d. &c.—*London,* 1839. 8vo. pp. 1408.

It will not be expected that we should devote much space to the notice of works which are probably, by this time, in the hands of most of our readers, and the preceding editions of which, at least, must be familiarly known to the whole profession. As in the exercise of our duty, however, we had to examine them, so are we bound, by the same obligation, to report the result of our examination. Works that are universally read or consulted have a fearful influence for good or evil; and it is our business to point out to our readers what they may seek and what they must eschew.

1. The Dictionary of Professor Cooper has been always regarded, both at home and abroad, as one of the most valuable productions of modern times. It has ever borne the impress of its author's estimable character, being marked by fulness, candour, and truth. The present edition is larger, by a sixth or seventh, than the preceding, and contains all the recent improvements that have been made known in this and other countries. The work is indeed, as it now stands, a complete encyclopædia of scientific and practical surgery, and is not merely suited to the young practitioner who has yet to learn his profession, but is fraught with so much recondite learning and practical information as to claim for it the attention of the most experienced surgeon. One conspicuous feature of this dictionary, its copious and accurate *Bibliography,* (which has been happily imitated, if not surpassed, by Dr. Copland,) renders it especially useful to all who are desirous of studying surgery extensively and as a science.

2. Dr. Hooper's Dictionary is of a different stamp, as to its plan and object; and it must be admitted to have been of very inferior execution, until it passed through the hands of its present editor. Now, indeed, it will yield to none of its competitors in point of excellence; and will stand, with them, a permanent memorial of the industry, learning, and talents of its authors: we say authors, because it will be found, on examination, that the work, as it now stands, is more the production of Dr. Grant than of Dr. Hooper. Indeed, had the editor been so minded, he might have placed his name first, if not singly, on the title-page, with fully as much propriety as a celebrated professor of the present day has blazoned his own to the occultation of the original author's. At any rate, whether he claims it or not, the merit we have indicated will be accorded to Dr. Grant by all who will take the trouble, as we have done, to compare the present edition with the former.

We consider the original plan of this dictionary essentially faulty, "inasmuch as it attempts to combine the objects of a technological lexicon with those of a compendious cyclopædia of the medical sciences." We regard the successful attainment of these two objects as impossible within the limits of a work of this sort, extensive as these now are. We think it would have been better to have left all *details* respecting the various branches of science, including medicine, to works of larger size, and specially devoted to this purpose, and to have constituted this volume a mere dictionary of terms, of definitions and derivations. We must admit, however, at the same time, with Dr. Grant, "that there must be something in its plan which is generally useful and adapted to the wants of a large class of readers;" else the previous editions could never have attained the very extensive circulation they have so long enjoyed. "The object of the editor, therefore, has been to improve it in its details, without altering the general principle of its construction;" and we can safely assure all those to whom the former editions were acceptable, that they will find this in every way greatly superior. Everything that was antiquated has been removed or renovated; a vast mass of errors, in the lexicographical part more especially, has been corrected; and the whole brought up to the level of our present knowledge. In accomplishing this important and very difficult task, Dr. Grant has evinced, in a striking degree, great talents, great industry, and accurate and extensive learning. Were not the volume before us so useful, we could almost regret that Dr. Grant had not engaged in some more original work, in accomplishing which his merits ran no risk of being lost in those of another.

